# Path analysis of influencing factors for anxiety and depression among first-trimester pregnant women

**DOI:** 10.3389/fpsyg.2024.1440560

**Published:** 2024-08-29

**Authors:** Wenjuan Li, Leshi Lin, Sheng Teng, Yi Yang, Li Li, Fang Peng, Dongmei Peng, Xiao Gao, Guojun Huang

**Affiliations:** ^1^Key Laboratory of Molecular Epidemiology of Hunan Province, School of Medicine, Hunan Normal University, Changsha, China; ^2^Changsha Hospital for Maternal & Child Health Care Affiliated to Hunan Normal University, Changsha, China; ^3^Hunan Institute for Tuberculosis Control (Hunan Chest Hospital), Changsha, China

**Keywords:** pregnant women, early pregnancy, anxiety, depression, path analysis, influencing factors

## Abstract

**Background:**

Prenatal anxiety and depression exert a significant influence on the physiological and psychological health outcomes of both expectant mothers and their infants. The aim of this study was to explore the intrinsic relationships between maternal anxiety, depression in early pregnancy, and their influencing factors. The findings of this study provide scientific basis for developing targeted preventive interventions.

**Methods:**

The study involved 887 expectant mothers in the early stages of pregnancy residing in Changsha City from March to August 2022. The sociodemographic characteristics, health and lifestyle factors, and pregnancy-related factors of participants were collected. The Edinburgh Postnatal Depression Scale and the Self-Rating Anxiety Scale were used to assess depression and anxiety, respectively. Chi-square tests and multivariate logistic regression models using SPSS 26.0 were used to assess factors impacting early pregnancy anxiety and depression. Amos 23.0 was used to construct a path model to determine the potential pathways of the influencing factors.

**Results:**

In early pregnancy, the prevalence of depression and anxiety were 17.4% and 15.8%, respectively. Path analysis showed that early pregnancy anxiety and illness during pregnancy had a direct effect on early pregnancy depression. Anxiety had the greatest overall impact on early pregnancy depression. Education, maternal comorbidities, symptoms of pregnancy, electronic device usage time, work stress, active smoking in the 6 months before pregnancy, and sleep quality were found to solely exert indirect effects on early pregnancy depression. Sleep quality had the greatest overall impact on early pregnancy anxiety. Active smoking in the 6 months before pregnancy, sleep quality, and work stress only had a direct impact on early pregnancy anxiety. Additionally, electronic device usage duration and monthly per capita household income exclusively indirectly impacted symptoms of early pregnancy anxiety.

**Conclusion:**

The study highlights the importance of targeted interventions in early screening. Avoiding excessive use of electronic devices and active smoking in the 6 months before pregnancy, alleviating work stress and symptoms of pregnancy, increasing education levels and monthly per capita household income, improving sleep quality, and actively preventing illnesses during pregnancy and maternal comorbidities might reduce anxiety and depression in early pregnancy.

## 1 Introduction

During pregnancy, women often face various psychological challenges that can adversely affect both their health and the long-term well-being of their fetus (Pampaka et al., 2018; Isaacs and Andipatin, 2020). Psychological health during pregnancy encompasses a range of emotions and states, including stress, fear, anxiety, depression, and sadness (Donegan et al., 2023). Prenatal anxiety usually refers to excessive and persistent worry experienced by pregnant women, often accompanied by symptoms such as restlessness, tension, and insomnia (Liu et al., 2024). Prenatal depression is characterized by sustained depressive moods during pregnancy, including sadness, loss of interest, fatigue, and sleep disturbances (Ayen et al., 2024). Anxiety and depression are the most common psychological symptoms related to pregnancy (Cena et al., 2021). During the coronavirus diseases 2019 (COVID-19) pandemic, a meta-analysis revealed a global pooled prevalence of 18.7% for anxiety and 25.1% for depression among expecting mothers. This underscored the prevalence and severity of these symptoms across different countries (Ghazanfarpour et al., 2022).

Studies have indicated that depression and anxiety during pregnancy may result in negative pregnancy outcomes, such as an increased risk of gestational diabetes and preeclampsia (Alawamir et al., 2017; Tang et al., 2020; OuYang et al., 2021), as well as heightened risks of spontaneous preterm birth, low birth weight, and stillbirth (Doktorchik et al., 2018; Chandra et al., 2021; Thomas et al., 2021). Beyond physical health impacts, prenatal psychological distress can severely affect maternal mental and emotional health, increasing the likelihood of postnatal anxiety and depression and potentially damaging the mother–infant bond (Dias and Figueiredo, 2015; Slomian et al., 2019; Obrochta et al., 2020), thereby intensifying the challenges faced by new mothers. For infants, these effects may extend to developmental challenges such as cognitive delays (Ibanez et al., 2015), neurodevelopmental issues (Sethna et al., 2021; O'Connor et al., 2022), behavioral risks (van den Bergh et al., 2006; van den Heuvel et al., 2018), difficulties in socioemotional development (Madigan et al., 2018), tendencies towards anxiety (Rifkin-Graboi et al., 2015), and depression (Pearson et al., 2013).

Many studies have revealed that expectant mothers undergo differing degrees of anxiety and depression throughout the different phases of pregnancy, necessitating period-specific measures to mitigate these conditions (Zhang et al., 2018). Research conducted during the pandemic identified early pregnancy as a predisposing factor for depressive and anxious symptoms in pregnant women (Wu et al., 2021). Furthermore, compared with the mid and late stages of pregnancy, the early trimester has higher screening rates for anxiety and depression (Miguez and Vazquez, 2021; Wenzel et al., 2021). These findings highlight that early pregnancy—the period spanning from conception to the 6th day of the 13th week (Al-Memar et al., 2020)—is a critical time for the psychological well-being of expectant mothers.

Despite many studies exploring the factors affecting anxiety and depression within the broader context of pregnancy and postpartum mental health trajectories, research specifically targeting the factors influencing early pregnancy depression and anxiety is still relatively limited. A significant issue is the comparatively lesser attention given to the early stages of pregnancy compared to other periods. In fact, only a few studies explicitly discuss and analyze the factors influencing anxiety and depression during early pregnancy. These factors include social support, pregnancy-related stress, sleep quality, noise exposure (Xuanyi et al., 2019), education level, employment status, pre-pregnancy nicotine use, history of depression or anxiety (Rubertsson et al., 2014), personal character traits, lack of spousal advice, level of family care, being a housewife or unemployed, primiparity, physical activity (Tang et al., 2019), not living with a partner, unintended pregnancy or prolonged conception time (van de Loo et al., 2018), low-income family function, low quality of life, and smoking history (Qin et al., 2023). These factors can be broadly classified as personal, family, and social. Although familial and societal factors have been extensively studied, the impact of individual health behaviors, such as electronic device usage time, work stress, and symptoms of pregnancy, remains unclear.

Previous studies on early pregnancy anxiety and depression primarily employed traditional regression statistical methods to study the influencing factors of one or two outcomes (Trost et al., 2016). These methods may not adequately capture the complex interactions and indirect effects among variables, making it challenging to fully understand the underlying mechanisms. Path analysis allows us to elucidate the properties and magnitude of path coefficients, clarifying the interrelationships between variables in the model. By employing path analysis, we can more accurately determine which variables have a direct impact on depression and anxiety, and which exert their effects indirectly through other mediating variables. This analytical approach not only enhances our understanding of the complexity of these mental health issues but also identifies potential risk factors (Liu et al., 2017).

This study used a path analysis model to explore the intrinsic relationships between maternal anxiety, depression in early pregnancy, and their influencing factors, focusing on sociodemographic, health, lifestyle, and pregnancy-related factors. This approach contribute to the development of targeted preventive interventions and provide a scientific basis for reducing anxiety and depression in early pregnancy.

## 2 Methods

### 2.1 Study participants

This study was conducted at Changsha Hospital for Maternal and Child Health Care from March to August 2022. It adhered to ethical standards, ensuring the rights and privacy of participants. All participants voluntarily enrolled, signed informed consent forms and retained the right to withdraw at any time without repercussions. Their personal information was securely stored and processed solely for the purposes of this study. The study was authorized by Hunan Normal University’s Ethics Committee (approval number: 2021 No. 278). Eligible participants were at least 18 years old, had a pregnancy duration of no more than 14 weeks, and provided consent to participate. Those with severe neuropsychiatric or organic diseases and incomplete questionnaire responses were excluded. Adequate sample size was determined using G*Power (Faul et al., 2007; Alzahrani et al., 2022). We set the alpha level at 0.05 and the power at 0.95, using a small effect size (*f* = 0.10) to detect significant results with 16 predictors tested. Consequently, the minimum required sample size was determined to be 298 participants. Throughout the study duration, 890 pregnant women participated; however, 3 participants were excluded due to incomplete data. Ultimately, 887 eligible women were enrolled in this study.

### 2.2 Data collection and procedure

This study was conducted at the obstetric outpatient clinic of Changsha Hospital for Maternal and Child Health Care. We utilized convenience sampling to distribute questionnaires randomly to pregnant women attending prenatal examinations, ensuring an equal selection probability for each participant. The investigators were uniformly trained and conducted face-to-face, one-on-one questionnaire interviews using standardized survey language. The questionnaire items were explained by the investigators, and participants filled them out on tablets based on their own circumstances. The survey collected maternal health information, including sociodemographic characteristics, health and lifestyle factors, and pregnancy-related factors.

### 2.3 Model construction

Our research employed the stress process model, which includes stressors, mediating factors, and stress outcomes (Ruyak et al., 2016). Stressors are any factors—whether biological, social, environmental or psychological—that challenge an individual’s ability to adapt or maintain balance. Mediators are variables that intervene in the relationship between stressors and stress outcomes. Stress outcomes are the end result of the interaction between stressors and mediators, reflecting the individual’s response to stress. In this study, education, early pregnancy symptoms, and pregnancy complications were identified as stressors. Behavioral, psychological, and health-related influencing factors were considered as mediating factors. Additionally, symptoms of anxiety and depression during early pregnancy were recognized as stress outcomes.

### 2.4 Research variables

The research variables included age (years) (<35 or ≥35), education (≤high school or ≥college), number of family inhabitants (≤2 or ≥3), occupation (employed or unemployed), medical insurance (yes or no), monthly per capita household income (RMB) (≤5,000 or >5,000), physical activity (low, moderate, or high), sleep quality (poor or very poor; average; or good or fairly good), nighttime sleep duration (h/day) (<8 or ≥8), maternal comorbidities (yes or no), unplanned pregnancy (yes or no), desired fetus sex (yes or no), active smoking in the 6 months before pregnancy (defined as women who had smoked more than 100 cigarettes in their lifetime and at least one cigarette per day in the 6 months before pregnancy) (yes or no), passive smoking in the 6 months before pregnancy (>15 min per day) (yes or no), active smoking in early pregnancy (defined as women who had smoked more than 100 cigarettes in their lifetime and at least one cigarette per day in early pregnancy) (yes or no), passive smoking in early pregnancy (>15 min per day) (yes or no), medication use in the 6 months preceding pregnancy (yes or no), illness during pregnancy (defined as physical illnesses) (yes or no), work stress (yes or no), parity (nulliparous or multiparous), and symptoms of pregnancy (any one of these symptoms: frequent urination, urgent urination, vomiting, abdominal pain, vulvar itching, leucorrhea, vaginal bleeding, and other symptoms) (yes or no). Pre-pregnancy body mass index (BMI) was categorized into three groups: underweight (BMI <18.5 kg/m^2^), normal weight (18.5 ≤ BMI <24.0 kg/m^2^), and overweight or obese (BMI ≥24.0 kg/m^2^). Electronic device usage time was defined as the average daily duration spent using electronic devices such as smartphones, tablets, and computers (≤4 h, ~8 h, ~12 h, or > 12 h).

### 2.5 Measures

The study employed William W. K. Zung’s Self-Rating Anxiety Scale (SAS), a 20-item tool using a four-point frequency scoring system to evaluate anxiety symptoms over the preceding week: “none or minimal time” (Level 1), “some of the time” (Level 2), “a considerable amount of time” (Level 3), and “the vast majority or all of the time” (Level 4) (Dunstan and Scott, 2020). Reverse scoring was applied to Items 5, 9, 13, 17, and 19. The whole raw score was normalized by multiplying by 1.25, with higher scores suggesting more acute anxiety. A standardized SAS score of 50 served as the cutoff for identifying early pregnancy anxiety in pregnant women, with scores of 50 or above indicating anxiety. The Chinese version of the SAS is commonly used by Chinese women during pregnancy. In our study, the internal consistency of the SAS, measured by Cronbach’s α coefficient, was 0.89 (Gao et al., 2014).

The Edinburgh Postnatal Depression Scale (EPDS) was developed to evaluate depression during pregnancy. The Chinese version of the EPDS has psychometric qualities that make it appropriate for evaluating perinatal depression (Hawley and Gale, 1998). It comprises 10 items, each scored on a scale from 0 to 3, reflecting the presence and severity of depressive symptoms, for a total possible score of 0–30 (Cox et al., 1987). Elevated scores indicate a greater severity of depressive symptoms. In our study, the threshold for depression during early pregnancy was set at an EPDS score ≥ 10. The Cronbach’s α coefficient for the EPDS was 0.876, and the test–retest reliability was 0.90 (Lau et al., 2010).

The International Physical Activity Questionnaire (IPAQ) assesses physical activity levels using a short form (Hagstromer et al., 2006). The form documents the weekly duration and frequency of walking, moderate, and vigorous activities, along with their corresponding metabolic equivalent (MET) values: 3.3 for walking, 4.0 for moderate activities, and 8.0 for vigorous activities. The calculation method for weekly MET minutes involved multiplying the MET value by the activity frequency and duration (in minutes). Overall activity levels are classified as high (≥1,500 MET-minutes/week from activities over ≥7 days), moderate (≥600 MET-minutes/week from activities over ≥5 days), or low (activities below these thresholds). The scale has been proven to have good reliability and validity (Chen et al., 2022).

### 2.6 Statistical analysis

Data were analyzed using SPSS 26.0 software. Statistical descriptions were conducted using measures such as mean, standard deviation, frequency, and percentages. The univariate analysis of categorical variables was conducted using chi-square tests. Anxiety and depression-related variables with *p*-values <0.1 were combined and used as independent variables in logistic regression analysis for both conditions. Furthermore, when early pregnancy depression was the dependent variable in the multivariate analysis, early pregnancy anxiety was added as a control variable in light of the cohabitation of anxiety and depression. The multivariate analysis results were then combined with path analysis to explore the relationships between variables and their associations with early pregnancy anxiety and depression. The model was examined using Amos 23.0. The model fit was continuously adjusted; eventually, it resulted in the formation of a path analysis model.

## 3 Results

### 3.1 Participants’ characteristics

Pregnant women (887) met the inclusion requirements. The average maternal age was 29.0 ± 4.1 years. Regarding sociodemographic characteristics, 82.6% of the women held a bachelor’s degree or higher. The average number of family inhabitants was 3.0 ± 1.1 persons. During pregnancy, 84.4% were employed, and 95.8% had health insurance. Most of these women (83.7%) reported a monthly household income per capita exceeding 5,000 yuan. Regarding health and lifestyle factors, 51.4% of women experienced work-related stress, and 90.1% engaged in low levels of physical activity. The average pre-pregnancy BMI was 21.3 ± 3.7 kg/m^2^ and nighttime sleep duration was 8.0 ± 1.1 h. Regarding sleep quality, 45.7% rated their sleep as average. Furthermore, 31.8% of the women used electronic devices for more than 8 h daily. Approximately 6 months before pregnancy, 5.2% were active smokers, and 47.9% were passive smokers. Simultaneously, 39.65% reported using medications, mainly cold remedies (25.6%) and antibiotics (7.4%). In the early stages of pregnancy, 1.1% were active smokers, while 33.1% were exposed to secondhand smoke. Upon analyzing pregnancy-related factors, we found that 19.5% of pregnant women had comorbidities. Unplanned pregnancies accounted for 39.9, and 26.4% had a preference for the sex of the fetus. A total of 16.8% of patients reported illnesses during pregnancy. Most participants (70.9%) were primiparous, and 84.4% had symptoms in early pregnancy, with the top three symptoms being vomiting (62.5%), frequent urination (44.5%), and increased vaginal discharge (21.6%). Additionally, 140 (15.8%) participants exhibited anxiety symptoms, and 154 (17.4%) exhibited depressive symptoms (Table 1).

**Table 1:** Univariate analysis of anxiety and depressive symptoms.

Variable	*N* (%)	No anxiety *n* (%)	Anxiety *n* (%)	χ^2^	*P*	No depression *n* (%)	Depression *n* (%)	χ^2^	*P*
Observations	887	747 (84.2)	140 (15.8)			733 (82.6)	154 (17.4)		
**Sociodemographic factors**									
Age (years)				0.090	0.764			0.981	0.322
<35	805 (90.8)	677 (90.6)	128 (93.4)			662 (90.3)	143 (92.9)		
≥35	82 (9.2)	70 (9.4)	12 (6.6)			71 (9.7)	11 (7.1)		
Education				25.314	**<0.001**			4.699	**0.030**
High school or below	154 (17.4)	109 (14.6)	45 (32.9)			118 (16.1)	36 (23.4)		
College or above	733 (82.6)	638 (85.4)	95 (7.1)			615 (83.9)	118 (76.6)		
Occupation				5.487	**0.019**			0.055	0.814
Employment	138 (15.6)	640 (85.7)	109 (75.7)			618 (84.3)	131 (85.1)		
Unemployment	749 (84.4)	107 (14.3)	31 (24.3)			115 (15.7)	23 (14.9)		
Medical insurance				2.119	0.145			0.488	0.485
No	37 (4.2)	28 (43.9)	9 (6.4)			29 (4.0)	8 (5.2)		
Yes	850 (95.8)	719 (96.3)	131 (93.6)			704 (96.0)	146 (94.8)		
Number of family inhabitants				2.791	**0.095**			0.132	0.717
≤2	380 (42.8)	329 (44.0)	51 (36.4)			312 (42.6)	68 (44.2)		
≥3	507 (57.2)	418 (56.0)	89 (63.6)			421 (57.4)	86 (55.8)		
Monthly per capita household income (RMB)				0.277	0.599			4.475	**0.034**
≤5000	145 (16.3)	120 (16.1)	25 (17.9)			111 (15.1)	34 (22.1)		
>5000	742 (83.7)	627 (83.9)	115 (82.1)			622 (84.9)	120 (77.9)		
**Health and lifestyle factors**									
Pre-pregnancy BMI				0.523	0.770			2.113	0.348
Underweight	148 (16.7)	124 (16.6)	24 (17.1)			121 (16.5)	27 (17.5)		
Normal	614 (69.2)	515 (68.9)	99 (70.7)			503 (68.6)	111 (72.1)		
Overweight/obese	125 (14.1)	108 (14.6)	17 (12.1)			109 (14.9)	16 (10.4)		
Work stress				2.767	**0.096**			0.022	0.883
No	431 (48.6)	372 (49.8)	59 (42.1)			357 (48.7)	74 (48.1)		
Yes	456 (51.4)	375 (50.2)	81 (57.9)			376 (51.3)	80 (51.9)		
Physical activity				1.026	0.599			3.054	0.217
Low	799 (90.1)	675 (90.4)	124 (89.2)			665 (90.7)	135 (87.7)		
Moderate	38 (4.3)	33 (4.4)	5 (3.6)			32 (4.4)	6 (3.9)		
High	49 (5.6)	39 (5.2)	10 (7.2)			36 (4.9)	13 (8.4)		
Sleep quality				33.853	**<0.001**			11.127	**0.004**
Poor/very poor	105 (11.8)	76 (10.2)	29 (21.4)			78 (10.6)	27 (17.5)		
Average	405 (45.7)	324 (43.4)	81 (57.9)			327 (44.6)	78 (50.6)		
Good/fairly good	377 (42.5)	347 (46.5)	30 (20.7)			328 (44.8)	49 (31.8)		
Nighttime sleep duration (hours)				0.009	0.923			0.342	0.559
<8	231 (26.0)	195 (26.1)	36 (25.7)			188 (25.6)	43 (27.9)		
≥8	656 (74.0)	552 (73.9)	104 (74.3)			545 (74.4)	111 (72.1)		
Medication use in the 6 months preceding pregnancy				8.632	**0.003**			6.496	**0.011**
No	536 (60.4)	467 (62.5)	69 (49.3)			457 (62.3)	79 (51.3)		
Yes	351 (39.6)	280 (37.5)	71 (50.7)			276 (37.7)	75 (48.7)		
Electronic device usage time (hours)				3.393	0.335			11.330	**0.010**
≤4	196 (22.1)	170 (22.8)	26 (18.6)			174 (23.7)	22 (14.3)		
~8	409 (46.1)	345 (46.2)	64 (45.7)			329 (44.9)	80 (51.9)		
~12	211 (23.8)	177 (23.7)	34 (24.3)			178 (24.3)	33 (21.4)		
>12	71 (8.0)	55 (7.3)	16 (11.4)			52 (7.1)	19 (12.3)		
Active smoking in the 6 months before pregnancy				16.362	**<0.001**			2.574	0.109
No	841 (94.8)	718 (96.1)	123 (87.9)			699 (95.4)	142 (92.2)		
Yes	46 (5.2)	29 (3.9)	17 (12.1)			34 (4.6)	12 (7.8)		
Passive smoking in the 6 months before pregnancy				1.191	0.275			0.046	0.830
No	462 (52.1)	395 (52.9)	67 (47.9)			383 (52.3)	79 (49.4)		
Yes	425 (47.9)	352 (47.1)	73 (52.1)			350 (47.7)	75 (50.6)		
Active smoking in early pregnancy				4.462	**0.035**			7.509	**0.006**
No	877 (98.9)	741 (99.2)	136 (97.1)			728 (99.3)	149 (96.8)		
Yes	10 (1.1)	6 (0.08)	4 (2.9)			5 (0.07)	5 (0.07)		
Passive smoking in early pregnancy				1.665	0.197			0.032	0.857
No	593 (66.9)	506 (67.7)	87 (62.1)			491 (67.0)	102 (66.2)		
Yes	294 (33.1)	241 (32.3)	53 (37.9)			242 (33.0)	52 (33.8)		
**Pregnancy-related factors**									
Symptoms of pregnancy				6.984	**0.008**			0.019	0.890
No	135 (15.2)	124 (6.6)	11 (7.9)			111 (15.1)	24 (15.6)		
Yes	752 (84.8)	623 (83.4)	129 (92.1)			622 (84.9)	130 (84.4)		
Illness during pregnancy				4.366	**0.037**			8.273	**0.004**
No	738 (83.2)	630 (84.3)	108 (77.1)			622 (84.9)	116 (75.3)		
Yes	149 (16.8)	117 (15.7)	32 (22.9)			111 (15.1)	38 (24.7)		
Parity				3.540	**0.060**			0.024	0.877
Nulliparous	629 (70.9)	539 (72.2)	90 (64.3)			519 (70.8)	110 (71.4)		
Multiparous	258 (29.1)	208 (27.8)	50 (35.7)			214 (29.2)	44 (28.6)		
Maternal comorbidities				11.665	**0.001**			7.164	**0.007**
No	714 (80.5)	616 (82.5)	98 (70.0)			602 (82.1)	112 (72.7)		
Yes	173 (19.5)	131 (17.5)	42 (30.0)			131 (17.9)	42 (27.3)		
Unplanned pregnancy				1.327	0.249			0.410	0.522
No	533 (60.1)	455 (60.9)	78 (55.7)			444 (60.6)	89 (57.8)		
Yes	354 (39.9)	292 (39.1)	62 (44.3)			289 (39.4)	65 (42.2)		
Desired fetus sex				3.590	**0.058**			0.076	0.782
No	653 (73.6)	559 (74.8)	94 (67.1)			541 (73.8)	112 (72.7)		
Yes	234 (26.4)	188 (25.2)	46 (32.9)			192 (26.2)	42 (27.3)		

BMI, body mass index. Values in bold represent statistical significance at the level of *p* < 0.1.

### 3.2 Analysis of factors influencing symptoms of anxiety and depression in early pregnancy

Univariate analysis showed that the relationships between anxiety symptoms and education, occupation, number of family members, work stress, sleep quality, maternal comorbidities, desired fetal sex, medication use in the 6 months preceding pregnancy, illness during pregnancy, parity, active smoking in the 6 months before pregnancy, symptoms of pregnancy, and active smoking in early pregnancy, was statistically significant (*p* < 0.1) (Table 1).

Additionally, depressive symptoms were significantly associated with education, monthly per capita household income, medication use in the 6 months preceding pregnancy, active smoking in early pregnancy, anxiety, illness during pregnancy, electronic device usage time, maternal comorbidities, and sleep quality (*p*< 0.1) (see Table 1).

Factors linked to anxiety and depression (*p*-values<0.1 in univariate analyses) were included in a multivariate logistic regression model. For exposure variables significantly related to anxiety, depression, or both in the multivariate analysis, the *p*-values, crude and adjusted odds ratios (ORs) (95% confidence interval [CI]) were reported.

Exposure factors significantly linked to a heightened risk of anxiety in early pregnancy included work stress (adjusted OR (aOR) = 1.82 [95% CI: 1.21–2.72]), maternal comorbidities (aOR = 1.88 [95% CI: 1.21–2.92]), illness during pregnancy (aOR = 1.66 [95% CI: 1.03–2.66]), active smoking in the 6 months before pregnancy (aOR = 3.57 [95% CI: 1.79–7.11]), and symptoms of pregnancy (aOR = 2.50 [95% CI: 1.27–4.93]). Conversely, higher education (college or above vs. high school or below; aOR = 0.33 [95% CI: 0.21–0.52]) and sleep quality (good or fairly good vs. poor or very poor; aOR = 0.24 [95% CI: 0.13–0.44]) were related to a decreased risk of anxiety in early pregnancy (Table 2).

**Table 2:** Multivariate logistic regression of influencing factors for anxious symptoms in early pregnancy (*n* = 887).

Variables	Univariate		Multivariate†	
	*OR* (95% CI)	*p*	*OR* (95% CI)	*p*
Education (high school or below)	1.00 (Reference)		1.00 (Reference)	
College or above	0.36 (0.24–0.54)	<0.001	0.33(0.21–0.52)	<0.001
Work stress (no)	1.00 (Reference)		1.00 (Reference)	
Yes	1.36 (0.95–1.96)	0.097	1.82(1.21–2.72)	0.004
Sleep quality (Poor/very poor)	1.00 (Reference)		1.00 (Reference)	
Average Good/fairly good	0.65 (0.40–1.07) 0.22 (0.13–0.40)	0.092	0.74 (0.44–1.26) 0.24 (0.13–0.44)	0.268 <0.001
Maternal comorbidities (no)	1.00 (Reference)		1.00 (Reference)	
Yes	2.01 (1.34–3.03)	0.001	1.88 (1.21–2.92)	0.005
Illness during pregnancy (no)	1.00 (Reference)		1.00 (Reference)	
Yes	1.59 (1.03–2.48)	0.038	1.66 (1.03–2.66)	0.037
Active smoking in the 6 months before pregnancy (no)	1.00 (Reference)		1.00 (Reference)	
Yes	0.27 (0.15–0.51)	<0.001	3.57 (1.79–7.11)	<0.001
Symptoms of pregnancy (no)	1.00 (Reference)		1.00 (Reference)	
Yes	2.33 (1.23–4.45)	0.010	2.50 (1.27–4.93)	0.008

^†^Multivariate logistic regression models included exposure variables related to anxiety and depression (*p*-value < 0.1, univariate analysis).

Factors significantly associated with an increased risk of depression in early pregnancy included anxiety in early pregnancy (aOR = 4.40 [95% CI: 2.93–6.61]), illness during pregnancy (aOR = 1.73 [95% CI: 1.11–2.69]), and excessive electronic device usage (~8 h, >12 h vs. ≤ 4 h; aOR = 1.90 [95% CI: 1.13–3.22], aOR = 2.62 [95% CI: 1.27–5.41]). The primary protective factor against depression in early pregnancy was high monthly per capita household income (*RMB*; >5,000 vs. ≤5,000; aOR = 0.63 [95% CI: 0.40–1.00]; Table 3).

**Table 3:** Multivariate logistic regression of influencing factors for depressive symptoms during early pregnancy (*n* = 887).

Variables	Univariate		Multivariate†	
	*OR* (95% CI)	*p*	*OR* (95% CI)	*p*
Anxiety in early pregnancy (no)	1.00 (Reference)		1.00 (Reference)	
Yes	4.60 (3.09–6.86)	<0.001	4.40 (2.93–6.61)	<0.001
Illness during pregnancy (no)	1.00 (Reference)		1.00 (Reference)	
Yes	1.83 (1.21–2.79)	0.004	1.73 (1.11–2.69)	0.016
Monthly per capita household income (RMB) ≤5,000	1.00 (Reference)		1.00 (Reference)	
>5,000	0.63 (0.41–0.97)	0.036	0.63 (0.40–1.00)	0.049
Electronic device usage time (≤4 h)	1.00 (Reference)		1.00 (Reference)	
~8 h ~12 h >12 h	1.92 (1.16–3.19) 1.46 (0.82–2.62) 2.89 (1.45–5.75)	0.011 0.195 0.002	1.90 (1.13–3.22) 1.41 (0.77–2.56) 2.62 (1.27–5.41)	0.016 0.263 0.009

†Multivariate logistic regression models included exposure variables related to anxiety and depression (*p*-value < 0.1, univariate analysis).

### 3.3 Path analysis of influencing factors for symptoms of anxiety and depression during early pregnancy

**Figure 1:**
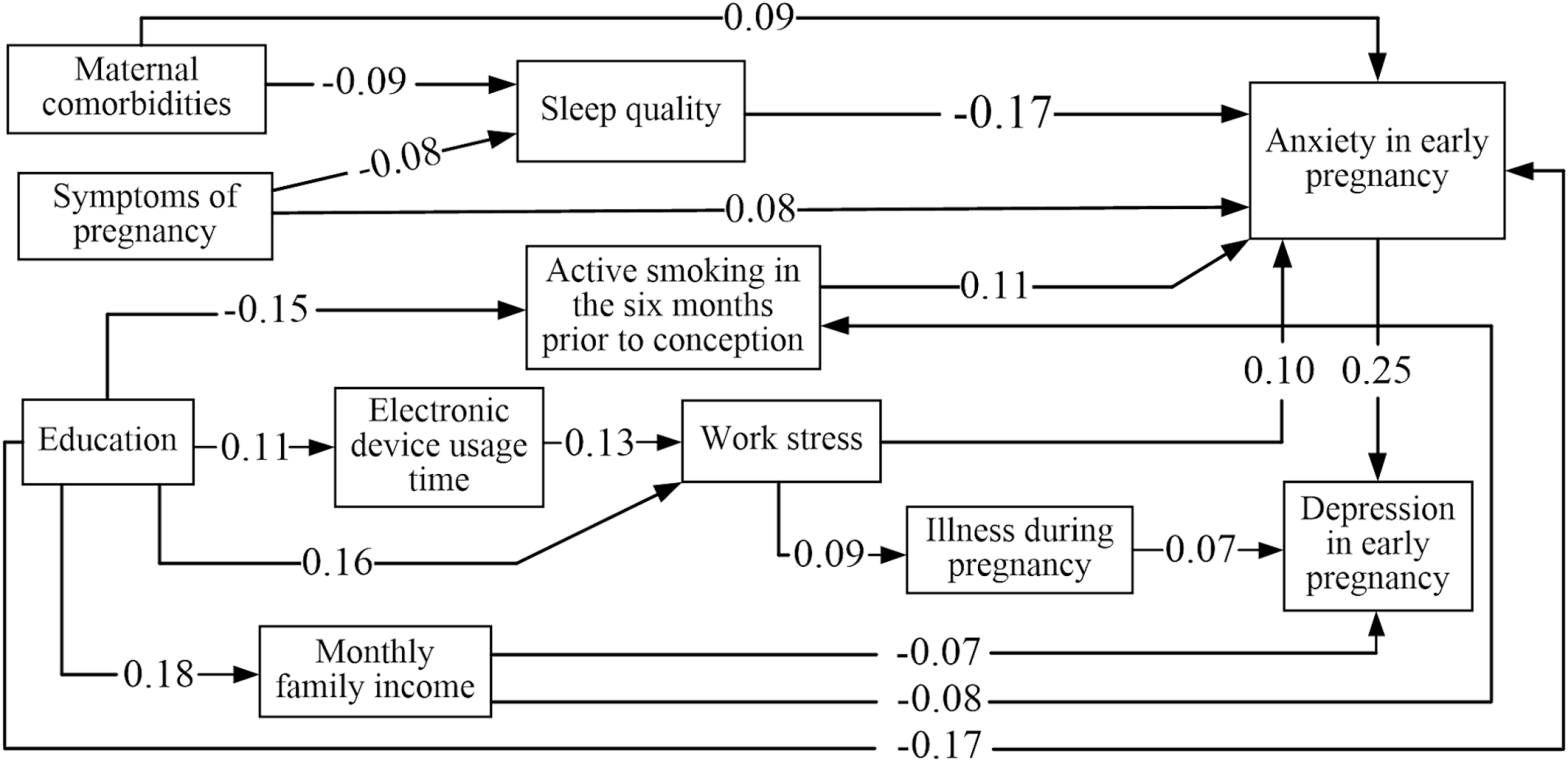
Path analysis of influencing factors for symptoms of anxiety and depression in early pregnancy.

The model depicted in Figure 1 includes only significant path coefficients. The standardized direct, indirect, and total impact estimates of depressive symptoms during early pregnancy are presented in Table 4. Monthly per capita household income exhibited both direct and indirect impacts on depression, with respective coefficients of-0.066 and-0.002, respectively. Anxiety had the greatest overall impact on depression, while the impact of being ill during pregnancy on depression was purely direct, with direct-effect coefficients of 0.075. Education, maternal comorbidities, symptoms of pregnancy, electronic device usage time, work stress, active smoking in the 6 months before pregnancy, and sleep quality were found to solely exert indirect effects on depressive symptoms. The coefficients for these indirect effects were 0.053, 0.027, 0.024, 0.004, 0.032, 0.028, and-0.044, in that order.

**Table 4:** Path analysis results of symptoms of anxiety and depression in early pregnancy.

Variables	Depression in early pregnancy			Anxiety in early pregnancy		
	Direct effect	Indirect effect	Total effect	Direct effect	Indirect effect	Total effect
Education	0.000	−0.053	−0.053	−0.167	−0.001	−0.168
Maternal comorbidities	0.000	0.027	0.027	0.090	0.016	0.107
Symptoms of pregnancy	0.000	0.024	0.024	0.082	0.015	0.097
Electronic device usage time	0.000	0.004	0.004	0.000	0.013	0.013
Monthly per capita household income	−0.066	−0.002	−0.068	0.000	−0.009	−0.009
Work stress	0.000	0.032	0.032	0.100	0.000	0.100
Active smoking in the 6 months before pregnancy	0.000	0.028	0.028	0.111	0.000	0.111
Sleep quality	0.000	−0.044	−0.044	−0.175	0.000	−0.175
Illness during pregnancy	0.075	0.000	0.075	0.000	0.000	0.000
Anxiety	0.253	0.000	0.253			

Additionally, four variables—education, symptoms of pregnancy, work stress, and maternal comorbidities—exerted both direct and indirect influences on anxiety. The overall impact coefficients amounted to-0.168, 0.097, 0.100, and 0.107, respectively. Active smoking in the 6 months before pregnancy, sleep quality, and work stress only had a direct impact on anxiety, with effect coefficients of 0.111, 0.100, and-0.175, respectively. Electronic device usage time and monthly per capita household income exclusively impacted anxiety symptoms indirectly, with effect coefficients of 0.013 and-0.009, respectively.

The fit results indicated that this path model was well fitted to the sample data (*p* = 0.176, CMIN/DF = 1.212, GFI = 0.991, NNFI = 0.942, IFI = 0.964, CFI = 0.961, AGFI = 0.984, and RMSEA = 0.015).

## 4 Discussion

Studies in prenatal mental health have progressively developed. However, research specifically focused on the critical period of early pregnancy is still insufficient. To our knowledge, this is the first study employing path analysis to explore the factors affecting symptoms of anxiety and depression in early pregnancy.

In this study, the prevalence rates of anxiety and depression among first-trimester pregnant women were 15.8% and 17.4%, respectively. The observed rate of early pregnancy anxiety (15.8%) aligns closely with findings from the Chinese Center for Disease Control and Prevention (16.02%) (Zhang, 2017) and a study conducted in Chongqing, China (15.04%) (Tang et al., 2019). Our study’s anxiety prevalence was close to the national average and slightly lower than the global prevalence of 18.2%, as reported in a comprehensive review of data from 34 countries (Dennis et al., 2017; Tang et al., 2019). Similarly, the early pregnancy depression rate of 17.4% in our study (17.4%) was comparable with that recorded during the COVID-19 pandemic in China (17.75%) (Qin et al., 2023). Moreover, the depression in our study rate likely falls within the national average for China (5.19%–36.4%) (Bödecs et al., 2009; de Jesus Silva et al., 2016; Tang et al., 2019; Smythe et al., 2022), but remains below the global prevalence in a review article (Ghazanfarpour et al., 2022). Most women in our sample were under 35, had higher education, stable employment, and health insurance, indicating a potentially lower risk of severe mental health outcomes. This low-risk status may imply that, although these women experienced significant anxiety and depression, their overall resilience might be higher compared to women from more disadvantaged backgrounds. Therefore, interventions for this population may need to focus more on stress management and support mechanisms rather than addressing socioeconomic vulnerabilities. Interestingly, the occurrence of depression during early pregnancy surpassed that of anxiety, which contrasts with the findings of the pre-pandemic research in China. For instance, a study in Chongqing among early pregnancy women reported that 15.04% experienced anxiety symptoms, whereas only 5.19% exhibited depressive symptoms (Tang et al., 2019). However, our findings are consistent with those of another study conducted among women in early pregnancy amid the COVID-19 pandemic in China, which reported depression rates of 17.75% and anxiety rates of 5.92% (Qin et al., 2023). This discrepancy may be attributed to the varying impacts of the COVID-19 pandemic on maternal mental health. A meta-analysis investigating the COVID-19 pandemic’s effects on perinatal health outcomes revealed a rise in maternal depression during the pandemic compared to pre-pandemic periods (Chmielewska et al., 2021), highlighting the need for greater focus and intervention in prenatal depression and anxiety.

Factors influencing anxiety in early pregnancy were categorized based on the absolute value of path coefficients. The order of influence from greatest to least was: sleep quality > education > active smoking in the 6 months before pregnancy > maternal comorbidities > work stress > symptoms of pregnancy > electronic device usage time > monthly per capita household income. Our findings demonstrated that physical activity and unplanned pregnancy did not significantly impact anxiety and depression during early pregnancy. These discrepancies with other studies may result from variations in screening tools, methodological choices, sample characteristics, and statistical analyses used (Miguez and Vazquez, 2021; Wenzel et al., 2021). Our study demonstrated that maternal comorbidities directly contributed to higher anxiety levels, consistent with previous research (Silva et al., 2017; Priyadarshanie et al., 2023). Maternal comorbidities also indirectly influenced early pregnancy anxiety by affecting sleep quality. This suggests the importance of enhancing screening and management of maternal comorbidities to ensure maternal and fetal health. Additionally, lower education levels were associated with increased anxiety symptoms through a direct pathway, which is consistent with earlier findings (Rezaee and Framarzi, 2014; Susanti et al., 2023). Education level also indirectly contributed to early pregnancy anxiety by influencing active smoking in the 6 months before pregnancy, monthly per capita household income, electronic device usage time, and work stress. Furthermore, symptoms such as vomiting and nausea during pregnancy were directly associated with anxiety in early pregnancy, aligning with previous studies (Beyazit and Sahin, 2018). These symptoms also indirectly affected early pregnancy anxiety through their impact on sleep quality. Prior research has shown that women experiencing more severe vomiting and nausea throughout pregnancy tend to have lower sleep quality, with more interruptions and awakenings at night (Laitinen et al., 2021). Sleep disturbances and anxiety during early pregnancy are linked to increased perinatal depression (King et al., 2022). There is a cross-sectional association between depressive and anxious symptoms and sleep disruptions (Polo-Kantola et al., 2017). Therefore, pregnant women experiencing these symptoms should seek support from healthcare professionals to manage physical discomfort and potential mental health impacts (Rubertsson et al., 2014).

Our study found that active smoking in the 6 months before pregnancy, sleep quality, and work stress directly impacted anxiety in early pregnancy. These factors played a significant mediating role between other variables and early pregnancy anxiety. Previous research indicated that women with depression and anxiety were more likely to smoke before pregnancy (Tong et al., 2016). Poor sleep quality increased anxiety symptoms in prenatal women (Yu et al., 2017). Prenatal work stress was associated with prenatal depression and anxiety (Clayborne et al., 2022). Based on these results, we recommend comprehensive interventions to reduce anxiety symptoms in early pregnancy. These measures could include enhancing health education, avoiding smoking, improving sleep quality, and implementing effective stress management and relief strategies to reduce anxiety risk.

Interestingly, we found that two factors—electronic device usage time and monthly per capita household income—only indirectly affected early pregnancy anxiety. Higher monthly per capita household income was associated with a lower likelihood of active smoking in the 6 months before pregnancy, subsequently reducing the risk of early pregnancy anxiety. Notably, a novel finding in our study was that electronic device usage time could indirectly influence early pregnancy anxiety through work stress. Increased electronic device usage time is linked to a higher risk of mental health issues, as individuals may be more prone to worry and anxiety, consistent with previous research (Lee and Cho, 2019). Therefore, pregnant women should use electronic devices in moderation and avoid prolonged continuous use.

The factors affecting depression during early pregnancy were categorized according to the absolute value of the path coefficient, with the degree of influence ranging from high to low: anxiety in early pregnancy > illness during pregnancy > monthly per capita household income > education > sleep quality > work stress > active smoking in the 6 months before pregnancy > maternal comorbidities > symptoms of pregnancy > electronic device usage time. In this study, anxiety and illness during pregnancy directly affected early pregnancy depression. Among the factors influencing early pregnancy depression, anxiety symptoms had the greatest impact, which is aligns with previous research results (Jiang et al., 2022). Maternal comorbidities, sleep quality, symptoms of pregnancy, education, active smoking in the 6 months before pregnancy, and work stress influenced depression through anxiety. Additionally, monthly per capita household income directly and indirectly influenced early pregnancy depression through active smoking in the 6 months before pregnancy and anxiety. Therefore, pregnant women with lower household incomes were more likely to experience prenatal depression, consistent with previous research (Sareen et al., 2011). Meanwhile, our path analysis indicated that the seven variables—education, maternal comorbidities, pregnancy symptoms, electronic device usage time, work stress, active smoking in the 6 months before pregnancy, and sleep quality—only influenced early pregnancy depression through indirect pathways. Specifically, these factors indirectly contributed to depression by influencing other variables, making them important underlying factors. The mother’s education level influenced early pregnancy depression by affecting monthly per capita household income. A study in Ethiopia showed a relationship between maternal depression and women’s education levels (Anato et al., 2020). Education level also impacted early pregnancy depression through work stress, electronic device usage time, and illness during pregnancy. Our study found that work stress was related to early pregnancy anxiety and could influence depression through anxiety and illness during pregnancy. Studies have indicated a link between prenatal work stress and anxiety and depression during and after pregnancy (Clayborne et al., 2022). Additionally, one study found that work stress can impact health through primary stress responses and mediators such as cortisol, which affect the immune, metabolic, cardiovascular systems, and brain homeostasis (McEwen and Stellar, 1993; McEwen and Gianaros, 2011). These findings may help develop more effective screening methods, prevention strategies, and interventions for the risks of anxiety and depression in the first trimester.

This study had two main strengths. First, we simultaneously included the two outcomes of anxiety and depression in early pregnancy, and applied path analysis for the first time to determine the potential causal paths of influencing factors of anxiety and depression in the first trimester. Second, we included factors related to the behavioral life of pregnant women, such as medication use in the 6 months preceding pregnancy, electronic device usage time, illnesses during pregnancy, and work stress.

However, this study has certain drawbacks. First, the participants were primarily enrolled from an urban population attending a maternity hospital, and the use of convenience sampling in this study may have introduced selection bias, potentially limiting the generalizability of the findings to broader populations. Additionally, the use of self-reported data and participants’ varying understandings of the research variables may have introduced information bias. Furthermore, other relevant factors, such as pre-existing mental health conditions and family support, were not considered, which could have led to confounding bias. Lastly, path analysis relies on the assumption that there are no unmeasured confounding factors, which may also introduce errors. Therefore, further studies with larger sample sizes from different regions and hospitals are necessary to provide stronger evidence. Future studies could also consider other factors such as pre-existing mental health conditions and family support.

## 5 Conclusion

Through path analysis, we identified potential influencing factors of anxiety and depression in early pregnancy and clarified their inherent interrelationships. Avoiding excessive use of electronic devices and active smoking in the 6 months before pregnancy, alleviating work stress and symptoms of pregnancy, increasing education levels and monthly per capita household income, improving sleep quality, and actively preventing illnesses during pregnancy and maternal comorbidities might reduce anxiety and depression in early pregnancy. In order to effectively prevent anxiety and depression in early pregnancy, it is crucial to promptly identify these influencing factors and implement corresponding preventive measures.

## Data Availability

The original contributions presented in the study are included in the article/supplementary material, further inquiries can be directed to the corresponding authors.
